# Cognitive control activity is modulated by the magnitude of interference and pre-activation of monitoring mechanisms

**DOI:** 10.1038/srep39595

**Published:** 2016-12-20

**Authors:** Jesús Cespón, Santiago Galdo-Álvarez, Fernando Díaz

**Affiliations:** 1 Cognitive Neuroscience Section, IRCCS Centro San Giovanni di Dio Fatebenefratelli, Brescia, (Italy); 2 Cognitive Neuroscience Laboratory, Facultade de Psicoloxía, Universidade de Santiago de Compostela, Santiago de Compostela (Spain)

## Abstract

The Simon task is used to study interference from irrelevant spatial information. Interference is manifested by longer reaction times when the required response –based on non-spatial features- is spatially incompatible with stimulus position. Interference is greater when incompatible trials are preceded by compatible trials (compatible-incompatible sequence) than when they are preceded by incompatible trials (incompatible-incompatible sequence). However, the relationships between spatial attention, interference and cognitive control have not been investigated. In the present study, we distinguished three experimental conditions according to sequential effects: same mappings (SM, compatible-compatible/incompatible-incompatible sequences: low interference), opposite mappings (OM, compatible-incompatible/incompatible-compatible sequences: high interference) and unrelated mappings (UM, central-compatible/central-incompatible sequences: intermediate interference). The negativity central contralateral (N2cc, a correlate of prevention of spatial response tendencies) was larger in OM than in SM, indicating greater cognitive control for greater interference. Furthermore, N2cc was larger in UM than in SM/OM, indicating lower neural efficiency for suppressing spatial tendencies of the response after central trials. Attentional processes (negativity posterior contralateral) were also delayed in UM relative to SM/OM, suggesting attentional facilitation by similar sets of attentional shifts in successive trials. Overall, the present findings showed that cognitive control is modulated by the magnitude of interference and pre-activation of monitoring mechanisms.

Cognitive control processes include a series of mechanisms (e.g. working memory, attentional control, inhibitory control, cognitive flexibility, problem solving and planning) that allow flexible and adaptive behaviour in response to environmental demands by implementing specific goal-directed actions^1^. Such abilities are crucial for carrying out daily life activities. Thus, unsurprisingly, these processes and the underlying neurophysiological mechanisms are considered a major topic in cognitive psychology and neuroscience^2^. The stimulus-response compatibility (SRC) paradigm is typically used in the study of cognitive control^3^. SRC tasks usually require inhibition of irrelevant salient stimulus features while attending to the relevant feature specified in task instructions, which indicates the required response.

The Simon task is an SRC task in which participants are required to respond to a non-spatial feature (e.g. colour, shape) of a lateralized stimulus by pressing one of two response buttons that are lateralized in the same spatial arrangement. Although stimulus position is irrelevant to task performance, the reaction time (RT) is longer when the response side is spatially incompatible with the stimulus position (incompatible stimulus-response (S-R) mappings) than in trials that require an ipsilateral response relative to stimulus position (compatible S-R mappings). The longer RT observed in incompatible trials reflects an interference effect known as the Simon effect (for a review see ref. 4). The Simon effect is related to activation of a prepotent response based on stimulus position, which delays the slower response based on the task instructions^5^,^6^. It has been suggested that an ancillary monitoring mechanism (AMM) inhibits the inappropriate spatial response and selects the response based on task instructions^7^. Therefore, in the Simon task, AMM represents a specific type of cognitive control involved in suppressing the prepotent tendency to respond towards the stimulus position, as it is irrelevant to task performance and can lead to erroneous responses.

The Simon effect is larger after a compatible than after an incompatible trial^8^,^9^. This type of modulation is known as the sequential effect and has also been reported in other SRC tasks^10^,^11^,^12^. The sequential effect was originally attributed to conflict adaptation^2^,^9^,^13^, although subsequent studies suggested a major role for episodic retrieval processes^12^,^14^,^15^; in other words, performance of an incompatible S-R trial activates a working memory trace that is retrieved when the next event is another incompatible S-R trial. However, when an incompatible S-R trial is preceded by a compatible S-R trial, the previously created trace must be inhibited, leading to longer RT. Identical reasoning may be applied to the compatible condition^16^.

Event-related potential (ERP) studies have focused on electrophysiological correlates of the Simon effect by employing the lateralized readiness potential (LRP)^17^. The LRP is obtained by a subtraction procedure that isolates an increase in activity at central electrode sites contralateral to the hand involved in preparing a movement. After applying the procedure, negative polarity waveforms indicate preparation of the correct response and positive polarity waveforms indicate preparation of the incorrect response. LRP peak latency constitutes a correlate of the overt response. Thus, LRP provides information about response preparation processes (for a review of LRP, see ref. 18). According to previously mentioned response competition models^5^,^6^, LRP studies suggested that the Simon effect occurs at the response selection stage^5^,^19^,^20^. Specifically, in incompatible trials, LRP shows an early and transitory positive dip related to response selection based on stimulus position. This early dip delays the onset of the negative polarity wave that is the correlate of response selection based on task instructions. Reduced interference in an incompatible trial has been attributed to a decreased positive dip^9^,^15^, which suggests attenuated strength of the response spatial tendency by sequential effects.

Attention to stimulus position and inhibition of the spatial tendency of the response are crucial processes in performance of a Simon task as the target stimulus is lateralized and the response spatial tendency must be inhibited. The negativity central contralateral (N2cc) is an ERP correlate of the cognitive control required to prevent response to spatial tendencies; in other words, N2cc is a correlate of the AMM. Visuospatial attention processes may also be studied by using the negativity posterior contralateral (N2pc).

The N2cc is obtained through a subtraction procedure that isolates an increase in activity at central electrode sites contralateral to the hemifield in which the target stimulus is located^21^,^22^. The N2cc is related to dorsal premotor (dPM) activity required to prevent cross-talk between the direction of spatial attention and manual response preparation^21^,^22^,^23^. A Simon task study demonstrated that N2cc was larger in the incompatible than in the compatible condition^24^. This finding was attributed to enhanced activity preventing the spatial tendency of the response and thus implementing the appropriate response in the incompatible condition. It therefore supported the view of N2cc as an AMM taking place during Simon-type tasks^7^,^25^.

The N2pc is obtained by applying the described N2cc procedure at parieto-occipital electrodes^26^,^27^,^28^. The N2pc has been related to visuospatial processing of target stimulus and inhibition of non-target stimuli^29^. Studies showed that the N2pc and concomitant visuospatial attention processes are not modulated by the Simon effect^23^,^24^,^30^,^31^. Importantly, inhibition of return (IOR), which can be defined as increased difficulty in re-allocating attention to the previously attended position^32^, modulates N2pc^33^ and may be an important variable when studying sequential effects in a Simon task.

Previous studies have not investigated modulation of attention to stimulus position or the cognitive control of response spatial tendency by sequential effects. Thus, in the present study we focused on those processes by considering N2pc and N2cc. As interference is modulated by sequential effects, it is reasonable to hypothesize concomitant modulation of cognitive control activity to manage the response spatial tendency; specifically, neural activity to prevent the response spatial tendency (whose ERP correlate is N2cc) should be higher at greater strength of conflict. Furthermore, studying sequential effects will allow determining whether the brain activity required to prevent response spatial tendencies is modulated according to the activation or non-activation of this cognitive control process in the previous trial. For visuospatial processes, it has been reported that spatial S-R compatibility tasks did not modulate N2pc. However, IOR may affect sequential effects, giving rise to N2pc modulations. Specifically, when a participant is responding to a lateralized stimulus (compatible or incompatible) preceded by a central stimulus, attention is always changed from the centre of the screen to a specific hemifield (i.e. to the right or the left hemifield). However, when participant is responding to a lateralized stimulus preceded by another lateralized stimulus, attention is reallocated to the same hemifield (and position) in half of the trials. Importantly, IOR can delay allocation of spatial attention and underlying brain activity (i.e. N2pc-related processes).

The task used in the present study comprised three types of experimental conditions according to the match between the current (n) and the previous (n-1) trial: same mappings (SM, compatible-Compatible (c-C) and incompatible-Incompatible (i-I) sequences), opposite mappings (OM, incompatible-Compatible (i-C) and compatible-Incompatible (c-I) sequences), and unrelated mappings (UM, neutral-Compatible (n-C) and neutral-Incompatible (n-I) sequences). N2cc and N2pc could not be obtained for neutral-neutral (n-N), compatible-neutral (c-N), and incompatible-neutral (i-N) sequences because the target stimulus was not lateralized. Nonetheless, these trials may provide additional support for IOR hypothesis and they were therefore included in several sets of RT analyses (for a graphic representation of types of trials and experimental conditions, see Fig. 1). SM and OM enabled study of cognitive control and visuospatial attention processes in conditions of low and high conflict respectively. Interestingly, UM enabled study of the cognitive control of the response spatial tendency after trials in which no such control was implemented because the target was located in the centre of the screen (i.e. n-I and n-C sequences). By contrast, attentional focus was always derived from a different location in UM (i.e. from the centre of the screen), whereas attention was re-oriented to the previously attended location in half of the SM and OM trials (as explained in Fig. 2), which may have resulted in IOR and concomitant modulation of N2pc, as argued in the previous paragraph. The use of specific behavioural analysis for the n-N, c-N, and i-N conditions enabled us to search evidence for IOR and to explore whether attending to a central position facilitates attending to an upcoming central position and also whether attending to a lateralized position facilitates attending to an upcoming lateralized position.

We predicted longer RTs in OM (i-C, c-I) than in UM (n-C, n-I) and SM (c-C, i-I) and in UM than in SM. Accordingly, longer LRP peak latencies were expected in OM than in UM/SM and in UM than in SM. Likewise, we predicted a larger N2cc amplitude in OM than in UM and SM, which would be consistent with implementation of greater cognitive control when interference is greater (Fig. 2, Hypothesis 1). However, absence of cognitive control when the target stimulus is located in the centre of the screen may lead to decreased neural efficiency in implementing cognitive control in a successive trial. In UM (n-C, n-I), the lateralized stimulus is preceded by a central stimulus (i.e. the target is located in the centre of the screen in the n-1 trial). Thus, a larger N2cc amplitude in UM than in SM (c-C, i-I) and OM (c-I, i-C) may be expected (Fig. 2, Hypothesis 2). Moreover, considering that attention always comes from a different position in UM but not in SM/OM, a longer N2pc latency may be expected in SM/OM than in UM due to IOR (Fig. 2, Hypothesis 3). According to the IOR hypothesis, attentional shift from the central to a lateralized spatial position (n-I and n-C trials) or vice versa (i-N and c-N trials) would be faster than reallocating attention to central (n-N trials) or lateralized positions (in half of the c-C, i-I, c-I, and i-C trials, the target at “n-1” and at “n” appeared at the same location). On the other hand, N2pc latency would be longer in UM than in SM/OM, assuming attentional facilitation from executing similar attentional sets of operations in two successive trials (SM and OM required a lateralized attentional shift, which also was preceded by another lateralized attended location) than in modified sets of operations (UM required a lateralized attentional shift, which was preceded by a centrally attended location) (Fig. 2, Hypothesis 4). According to this hypothesis, the RTs would be faster in trials in which the current “n” and the previous “n-1” targets are both central (n-N) or lateralized (c-C, i-I, c-I, and i-C) (i.e. similar set of attentional operations in “n” and “n-1”) than in trials in which the attentional focus changes from central-to-lateralized (n-I and n-C trials) or from lateralized-to-central (i-N and c-N trials) locations (i.e. different set of attentional operations in “n” and “n-1”).

## Method

### Participants

The study, which received prior approval by the local ethical review board, involved 17 participants (13 women) between 21 and 29 years old. Fifteen of the participants were right-handed and two were left-handed, as evaluated by the Edinburgh Handedness Inventory^34^. The participants had normal or corrected to normal vision and none had any history of neurological or psychiatric disorders. The present research was performed in accordance with the ethical guidelines laid down in the 1964 Declaration of Helsinki. The experimental protocols received prior approval by the University of Santiago de Compostela (USC) ethical committee. The experimental procedures were carefully explained to all the participants, who volunteered to take part in the study. Informed consent was obtained from all participants.

### Task and procedure

**Figure 1:**
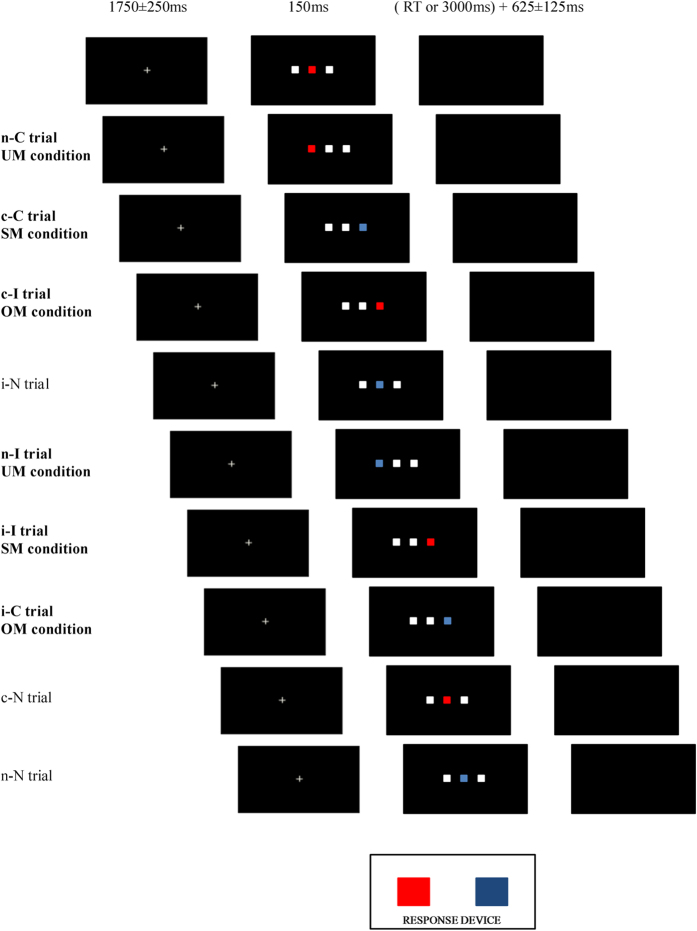
Graphical representation of trials and experimental conditions: Unrelated Mappings condition (UM) comprised a compatible stimulus preceded by a central stimulus (n-C trials) or an incompatible stimulus preceded by a central stimulus (n-I trials); Same Mappings condition (SM) comprised a compatible stimulus preceded by another compatible stimulus (c-C trials) or an incompatible stimulus preceded by another incompatible stimulus (i-I trial); and the Opposite Mappings condition (OM) comprised an incompatible stimulus preceded by a compatible stimulus (c-I trials) or a compatible stimulus preceded by an incompatible stimulus (i-C). The trials used and the corresponding experimental conditions are highlighted in bold type.

During the task, three squares each of 1.7 cm appeared in a horizontal arrangement on the screen (see Fig. 1). One square appeared in blue or in red (target stimulus) while the other two squares (non target stimuli) appeared in grey. The screen was placed 100 cm in front of the participants and each stimulus therefore occupied 0.67° of the visual angle. The space between squares was 0.72° and the complete display occupied 3.45°. The three squares were projected on the foveal region (see ref. 35). Each trial started with a grey central fixation cross of 0.36° × 0.36°, which was presented for 1750 ± 250 ms. The squares were then displayed in the centre of the screen, against a black background, for 150 ms. The screen then remained blank (black) until the participant responded (or for 3000 ms in case of no response). The screen then remained blank for another 625 ± 125 ms before the start of a new trial.

Participants were instructed to direct their gaze to the centre of the screen throughout the task and to respond to the colour of the target by pressing one of two buttons (also arranged horizontally) with the corresponding hand. In the example shown in Fig. 1, appearance of the red square required the left button to be pressed with the left hand and appearance of the blue square required the right button to be pressed with the right hand (response buttons were counterbalanced between participants). The trials were divided into compatible (C), incompatible (I) and neutral (N) according to the compatibility between the stimulus position and the side of the required response. Considering these three types of trials in relation to the previously presented trial, 9 different types of sequence were distinguished: compatible-Compatible (c-C), incompatible-Compatible (i-C), neutral-Compatible (n-C), incompatible-Incompatible (i-I), compatible-Incompatible (c-I), neutral-Incompatible (n-I), compatible-Neutral (c-N), incompatible-Neutral (i-N), neutral-Neutral (n-N) (capital letters indicate the current trial and lower case letters indicate the previous trial).

After a practice block of 18 trials, 720 trials (80 per each type of sequence) were presented at random in eight blocks (90 s inter-block interval). The experimental conditions of the present study were created by grouping the trials as follows: c-C + i-I trials (trials with same S-R mappings: SM); i-C + c-I trials (trials with opposite S-R mappings: OM); and n-C + n-I trials (trials with unrelated S-R mappings: UM). The probability of trial appearance was matched between conditions as performance may be modulated by differences in stimulus appearance probability (i.e. by oddball-like designs)^36^.

### EEG recordings

The following 49 active electrodes were used during the EEG recordings (Easycap, GmbH, Brain Products) in accordance with the 10–10 International System: AFz, AF3, AF4, AF7, AF8, Fz, F3, F4, F5, F6, F7, F8, FCz, FC1, FC2, FC3, FC4, FT7, FT8, FT9, FT10, Cz, C1, C2, C3, C4, C5, C6, T7, T8, CPz, CP3, CP4, TP7, TP8, TP9, TP10, Pz, P3, P4, P7, P8, P9, P10, PO7, PO8, Oz, O1 and O2. The EEG signal was passed through a 0.01–100 Hz analog bandpass filter and was sampled at 500 Hz. The reference electrode was placed on the tip of the nose and the ground electrode at Fpz. Recordings of vertical ocular movements (VEOG) and horizontal ocular movements (HEOG) were obtained with two electrodes located supra- and infraorbitally to the right eye and two electrodes at the external canthus of each eye, respectively. Impedance was maintained below 10 kΩs. After signal storage, ocular artefacts were corrected by independent component analysis. The signal was filtered at 0.01–30 Hz digital band-pass. Epochs exceeding ±100 μV were automatically rejected, and all the remaining epochs were individually inspected to identify those still displaying artefacts, which were also excluded from subsequent averaging. Epochs were then corrected to the mean voltage of the 200 ms pre-stimulus recording period (baseline).

### Data analysis

Trials with incorrect responses were excluded from analyses. Considering the mean reaction time (RT) and the corresponding distributions, RTs outside the 100–1000 ms range were considered outliers and were excluded from subsequent analyses, a range usually selected in previous studies^30^,^37^,^38^.

The epochs were established between −200 and 700 ms relative to the onset of the target stimulus. Following previous studies^39^, a two-step procedure was used to eliminate epochs with horizontal ocular artefacts. Specifically, trials with horizontal eye movements larger than ±35 μV were removed. Averaged HEOG waveforms showing residual eye movements exceeding ±3 μV within the 0–500 ms time window were also eliminated. Five participants (four women) were excluded from further analyses because they exhibited residual horizontal ocular movements in one or more conditions. The number of averaged epochs in each experimental condition was as follows: SM (108 epochs), OM (101 epochs) and UM (105 epochs).

The N2pc component was determined in relation to the hemifield of target presentation, as follows: [PO8 − PO7 (left hemifield) + PO7 − PO8 (right hemifield)]/2 (see ref. 27). The same formula was used for N2cc, in C3/C4 electrodes pair as follows: [C4 − C3 (left hemifield) + C3 − C4 (right hemifield)]/2 (see ref. 22). Peak latency values were measured as the time points at which the N2pc and N2cc reached the maximum negative amplitudes. N2cc amplitudes were calculated as average voltage between 200 and 270 ms (after grand average inspection). However, as N2pc showed differences in latency between conditions, the N2pc peak amplitudes were calculated as average amplitude in an 80 ms time window around peak latency for each participant.

The stimulus-locked lateralized readiness potential (sLRP) was calculated as contralateral-ipsilateral differences in activation for the primary motor cortex in each hemisphere. The differences were then averaged^40^. The method can be summarized by the formula [C4 − C3 (left hand movements) + C3 − C4 (right hand movements)]/2. Deflections with negative polarity indicate correct preparation of the correct response. The onset latency of the correct preparation in the sLRP was determined by the method of Schwarzenau *et al*.^41^, which assumes that the onset of correct preparation corresponds to the intersection point of two straight lines, one fitted to the baseline and another to the rising slope of the LRP. The sLRP peak latency was measured as the largest negative peak between 300 and 500 ms after stimulus presentation.

The study of N2cc and LRP in Simon-type tasks distinguishing compatible and incompatible conditions is complicated because an N2cc/LRP overlap takes place in central brain regions, which precludes reliable, separate measurement of each component^42^. However, this was not a problem in the present study because all the waveforms were equally compounded by spatially compatible and incompatible conditions, thereby removing N2pc/N2cc from LRP waveforms and LRP from N2pc and N2cc waveforms^43^,^44^.

### Statistical analysis

#### Behavioural analysis for testing sequential effects

The experimental conditions highlighted in bold in Fig. 1 were included in order to test the predicted sequential effects. Reaction time (RT) and number of errors (NE) were analyzed using the corresponding repeated measures ANOVA with two within-subject factors: Condition (two levels: Compatible, Incompatible) and type of sequence or mapping (three levels: SM, OM, UM). By distinguishing Condition and Type of sequence factors, this analysis allowed us to rule out a possible effect of the Condition × Sequence interaction, which may indicate differences between c-C and i-I in the SM condition, between n-C and n-I in the UM condition and/or between i-C and c-I in the OM condition.

#### Behavioural analysis for testing Inhibition of Return (IOR)

This analysis enabled RTs to be tested in trials in which attentional reallocation (and possible IOR) occurred in comparison with RTs in trials in which attention was changed from one to another location. The analysis included trials with the following sequences: c-C, i-I, c-I, and i-C. In half of these sequences, attention is directed to the same position in “n” relative to “n-1” trial. In the other half of c-C, i-I, c-I, and i-C sequences, attention is changed to contralateral hemifield in “n” relative to “n-1” trial. RTs were analyzed by using a repeated measures ANOVA with two within-subject factors: Attentional direction (two levels: attentional change, attentional reallocation) and Type of mapping (two levels: SM, OM). UM was not included in this ANOVA because all UM trials involve an attentional change in “n” relative to the “n-1” trial.

#### Behavioural analysis for testing different sets of attentional shift operations

This analysis aimed to test a possible facilitation effect from similar sets of attentional shift operations in two successive trials. The analysis included the nine types of sequences in order to investigate whether lateralized-to-lateralized (c-C, i-I, c-I, i-C) and central-to-central (n-N) attentional shifts (which involved similar sets of attentional operations in “n” and “n-1”) were faster than central-to-lateralized (n-C, n-I) and lateralized-to-central (i-N, c-N) attentional shifts (which involved different sets of attentional operations at “n” and “n-1”). RTs were analyzed by a repeated measures ANOVA with two within-subject factors: Current attentional focus (two levels: lateralized target, central target) and Previous attentional focus (two levels: same, different).

#### Event-related potential analysis

Event-related potentials (i.e. peak latencies of N2pc, N2cc and LRP, onset latency of LRP and amplitudes of N2pc, N2cc and LRP) were analyzed using the corresponding repeated measures ANOVA with a within-subject factor, Sequence (three levels: SM, OM and UM). A Greenhouse-Geisser correction for the degrees of freedom was performed when the condition of sphericity was not met. In these cases, the corresponding degrees of freedom are provided. For significant results, measures of size effect are also provided as the partial eta square (*η*^2^_p_). When the ANOVAs revealed significant effects due to the factors and their interactions, post hoc comparisons of the mean values were carried out by paired multiple comparisons (with Bonferroni correction).

## Results

### Behavioural results

#### Behavioural analysis for testing sequential effects

The repeated measures ANOVA (Condition × Sequence) for Reaction Times (RT) revealed that the Condition factor exerted a significant effect (F (1, 11) = 137.2, p < 0.001, η^2^_ρ_ = 0.926). RT was longer in the Incompatible than in the Compatible condition (p < 0.001). The Sequence factor also revealed a significant effect (F (1.17, 12.9) = 16.2, p = 0.001, η^2^_ρ_ = 0.605). RT was longer in OM than in SM (p = 0.003) and UM (p = 0.010) conditions. The RT was also longer in the UM than in the SM condition (p = 0.012). The Condition × Sequence interaction effect was not significant (F (2, 22) = 0.685, p = 0.514, η^2^_ρ_ = 0.059).

The repeated ANOVA (Condition × Sequence) for number of errors (NE) revealed a significant effect of the Condition factor (F (1, 11) = 16.1, p = 0.002, η^2^_ρ_ = 0.595), as the NE was higher in the Incompatible than in the Compatible condition (p = 0.002). The Sequence factor revealed a statistically significant effect (F (1.3, 14.9) = 7.0, p = 0.013, η^2^_ρ_ = 0.389), as the NE was higher in OM than in UM (p = 0.012) and marginally higher in OM than in SM (p = 0.059). The Condition × Sequence interaction effect was not significant (F (2, 22) = 0.443, p = 0.648, η^2^_ρ_ = 0.039).

#### Behavioural analysis for testing Inhibition of Return (IOR)

The repeated measures ANOVA (Attentional direction × Type of mapping) revealed a significant effect for Type of mapping (F (1, 11) = 29.37, p < 0.001, η^2^_ρ_ =  0.728), as RTs were faster in SM than in OM (p < 0.001). The Attentional direction factor did not reveal a significant effect (F (1, 11) = 0.547, p = 0.475, η^2^_ρ_ = 0.047). The Attentional direction × Type of mapping interaction did not reveal any significant effect (F (1, 11) = 2.163, p = 0.169, η^2^_ρ_ = 0.164).

#### Behavioural analysis for testing different sets of attentional shift operations

The repeated measures ANOVA (Current attentional focus × Previous attentional focus) revealed a significant effect for Current attentional focus (F (1, 11) = 15.4, p = 0.002, η^2^_ρ_ = 0.584), as RT was faster when attention was focused on the central position than on lateralized positions (p = 0.002). The factor Previous attentional focus also revealed a significant effect (F (1, 11) = 11.6, p = 0.006, η^2^_ρ_ = 0.513), as RT was slower for central-to-lateralized/lateralized-to-central than for central-to-central/lateralized-to-lateralized attentional changes (p = 0.006). The Current attentional focus × Previous attentional focus revealed a significant effect (F (1, 11) = 14.1, p = 0.003, η^2^_ρ_ = 0.562): RT was faster when attention was reallocated to the centre of the screen (central-to-central sequence) than in trials in which attention is reallocated to a lateralized position (lateralized-to-lateralized sequence) (p = 0.001).

### Electrophysiological results

For N2pc (see Fig. 3) latency, the repeated measures ANOVA (Sequence) revealed a significant effect of the condition or type of Sequence (F (2, 22) = 12.1, p < 0.001, η^2^_ρ_ = 0.524), as N2pc peak latency was longer in UM than in SM (p = 0.006) and OM (p = 0.004). For the N2pc amplitude, the repeated measures ANOVA (Sequence) did not reveal any significant effect (F (2, 22) = 1.041, p = 0.370, η^2^_ρ_ = 0.086).

For N2cc (see Fig. 4) latency, the repeated measures ANOVA (Sequence) did not reveal any significant effects (F (2, 22) = 1.851, p = 0.181, η^2^_ρ_ = 0.144). By contrast, for the N2cc amplitude, the repeated measures ANOVA (Sequence) revealed a significant effect of the Sequence factor (F (2, 22) = 20.57, p < 0.001, η^2^_ρ_ = 0.652), as the N2cc amplitude was larger in UM than in SM (p < 0.001) and OM (p = 0.008). N2cc was also larger in OM than in SM (p = 0.045).

For LRP (see Fig. 4) peak latency, the repeated measures ANOVA (Sequence) revealed an effect of the Sequence factor (F (1.32, 14.53) = 15.36, p < 0.001, η^2^_ρ_ = 0.583), as the LRP peak latency was longer in the OM than in the SM and UM conditions. It was also longer in UM than in SM (p = 0.041). For LRP onset latency, the repeated measures ANOVA (Sequence) did not reveal any significant effect (F (2, 22) = 2.503, p = 0.105, η^2^_ρ_ = 0.185).

## Discussion

Behavioural (RT and NE) and electrophysiological (LRP) data revealed greater interference in opposite mappings than in same mappings and unrelated mappings. The findings showed that N2cc was modulated by both the magnitude of interference (Hypothesis 1: larger N2cc in OM than in SM) and pre-inactivation of monitoring mechanisms (Hypothesis 2: larger N2cc in UM than in SM). Furthermore, N2cc was larger in UM than in OM, suggesting stronger N2cc modulation by pre-inactivation of monitoring mechanisms than by magnitude of interference. Negativity posterior contralateral (N2pc, correlate of visuospatial attention) was slower in UM than in SM and OM. Thus, the results did not support the hypothesis based on inhibition of return but were consistent with the predictions about different attentional operation sets (Hypothesis 4). This pattern of N2pc results was consistent with the RT results; specifically, RT was faster in central-to-central and lateralized-to-lateralized than in central-to-lateralized and lateralized-to-central sequences (for a summary of results and significant differences between conditions, see Table 1; the main hypotheses and related results are summarised in Fig. 5).

RT was longer in OM than in SM and UM, and it was longer in UM than in SM. Furthermore, NE was higher in OM than in SM and UM. In the framework of the theory of event code^16^, responses that are slower and more prone to error in OM reflect greater effort and time required to suppress the preceding stimulus-response (S-R) mapping, which was retrieved in working memory when the second trial of the sequence was presented. However, the matching between current and previous S-R mappings led to a shorter RT in SM. Previous S-R mapping was not retrieved in UM because current and previous S-R mappings do not share the same spatial properties. Therefore, as neither interference nor facilitation occurred, the RT was intermediate. Behavioural analyses did not show any different sequential effects for compatible or incompatible trials. Thus, averaging compatible and incompatible trials in ERP analyses did not involve loss of information regarding neural correlates of behavioural sequence effects. Thus, merging spatially compatible and incompatible conditions for ERP analyses seems an acceptable strategy for handling residual motor activity in N2cc and N2pc waveforms as well as residual N2pc/N2cc-related activity in LRP waveforms (for a review of N2pc/N2cc/LRP overlaps see ref. 42). On the other hand, RT results were not consistent with IOR taking place during the designed Simon-type task, as RTs were not longer when attention in the “n” trial was reallocated to the “n-1” position. Thus, the pattern of RTs provided support for facilitation during execution of similar attentional operation sets in two successive trials, as the responses were faster in central-to-central and lateralized-to-lateralized sequences (which involve similar operation sets at “n” and “n-1” positions, i.e. attending to a central and then to another central stimulus or attending to a lateralized and then to another lateralized stimulus) than in central-to-lateralized and lateralized-to-central sequences.

Consistent with RT, the LRP peak latency was longer in OM than in UM and SM, and it was longer in UM than in SM. Differences in LRP peak latency, but not in LRP onset latency, suggest differences in the speed of response execution. In contrast to previous findings^9^,^15^, no transitory LRP positive dip related to preparation of the incorrect response was observed. It is possible that the positive dip was jittered in the present study by averaging incompatible and compatible trials. However, it is also plausible that in previous studies the positive dip was related to N2cc and not to preparation of the incorrect response. Spapé *et al*.^15^ presented the stimuli in a horizontal arrangement and averaged compatible and incompatible trials separately, which produces N2cc/LRP overlap^42^. Although Stürmer *et al*.^9^ presented stimuli in a vertical arrangement, the N2cc may still affect LRP waveforms, as suggested by Praamstra^42^ and demonstrated in a later study^19^.

**Figure 2:**
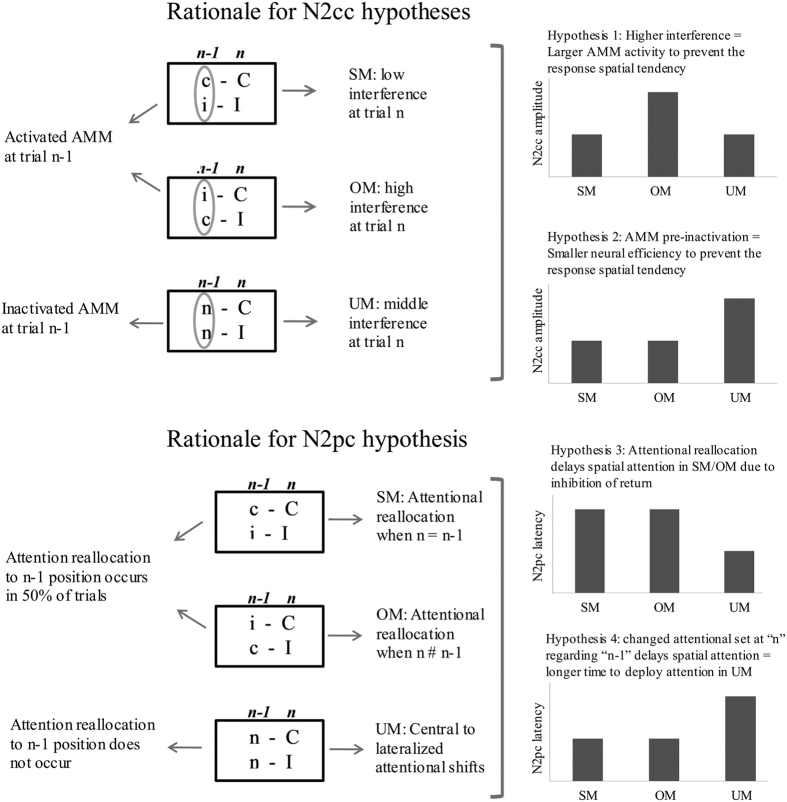
Hypotheses for cognitive control of the response spatial tendency (whose correlate is the negativity central contralateral -N2cc) and visuospatial attention (whose correlate is the negativity posterior contralateral -N2pc) modulations. AMM: Ancillary monitoring mechanism; n: current and analyzed trial; n-1: trial preceding the analyzed trial. SM: Same Mappings condition (c-C, i-I); OM: Opposite Mappings condition (i-C, c-I); UM: Unrelated Mappings condition (n-C, n-I).

**Figure 3:**
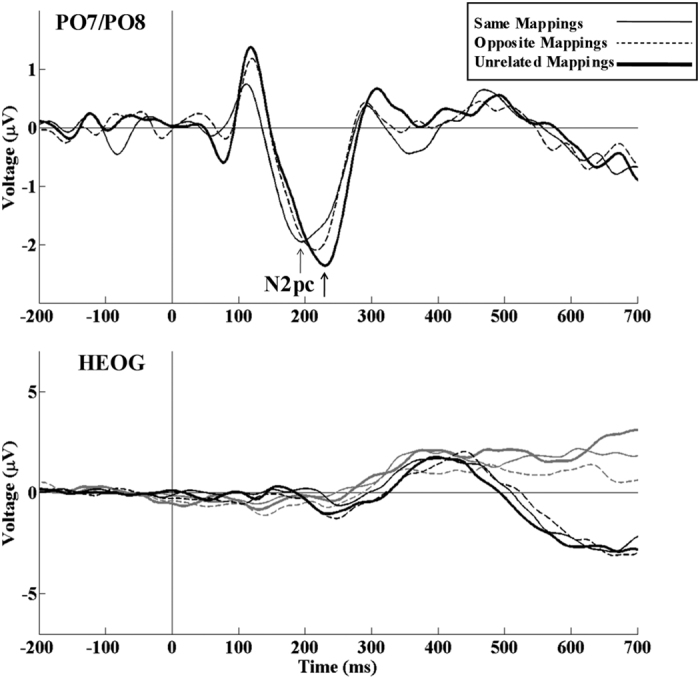
Modulations of visuospatial attention correlate. The negativity posterior contralateral (N2pc) obtained at PO7/PO8 was longer in UM than in SM and OM. Measurement of horizontal electrooculographic activity (HEOG) did not reveal any differences between experimental conditions.

**Figure 4:**
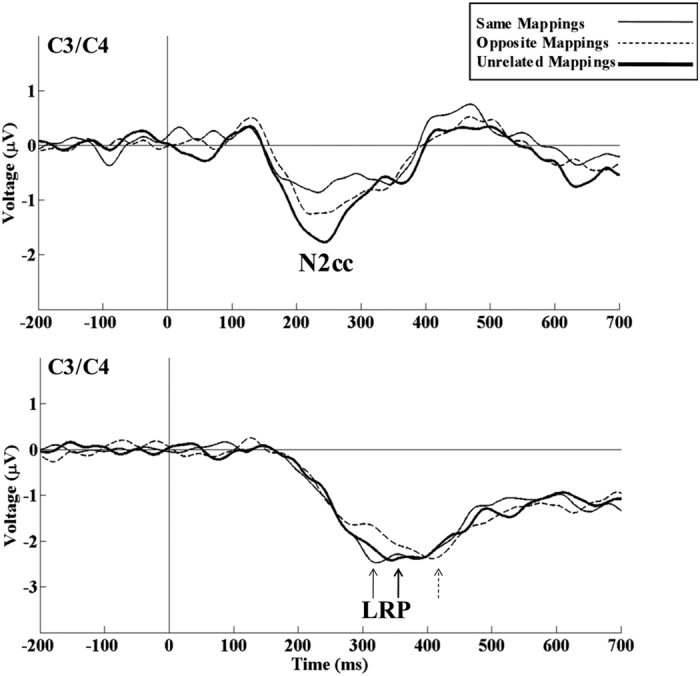
Modulations of interference and cognitive control correlates. Negativity central contralateral (N2cc) and lateralized readiness potential (LRP) at C3/C4 (see the corresponding formula in the main text). N2cc was larger in UM than in OM and SM and in OM than in SM. The LRP onset latency indicated differences between conditions. The peak LRP latency was longer in OM than in UM and SM, and it was longer in UM than in SM.

**Figure 5:**
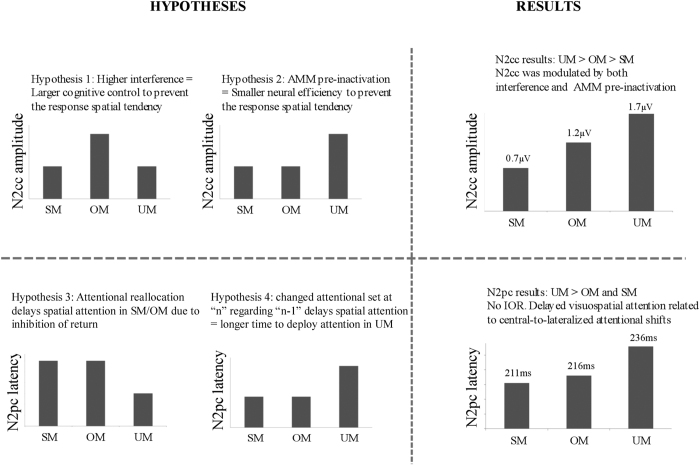
N2pc and N2cc hypotheses and results. This Figure graphically represents the main hypotheses (the rationale of which is explained in Fig. 2) and the related results; specifically, larger N2cc amplitude in UM than in OM and SM and larger N2cc in OM than in SM; longer N2pc latency in UM than in SM and OM conditions.

The main aim of the present study was to investigate how sequential effects modulate the activity underlying cognitive control. The N2cc component is a correlate of dPM activity^21^,^22^,^23^ related to an ancillary monitoring mechanism (AMM) involved in preventing response spatial tendencies during Simon-type tasks^7^,^25^. The results of the present study revealed a larger N2cc amplitude in OM than in SM. Therefore, the greater interference in OM was related to greater implementation of cognitive control mechanisms in OM than in SM, which is consistent with Hypothesis 1 for N2cc (Fig. 2). The relationship between increased interference and greater cognitive control activity is consistent with the findings of a previous Simon task study reporting larger N2cc amplitude in the incompatible than in the compatible condition^24^.

The N2cc was larger in UM than in OM and SM. According to Hypothesis 2 for N2cc (Fig. 2), this may be explained by decreased neural efficiency in UM, which would be related to pre-activation of N2cc neural sources (dPM) in SM and OM but not in UM. As the n-1 stimulus was lateralized in SM and OM, dPM was activated in these trials, thus enhancing the neural efficiency for implementing this activity in a subsequent trial. By contrast, the n-1 stimulus was centrally located in UM, and dPM was not activated in these trials as it was not necessary to monitor the spatial response to a central target. Overall, although RT is faster in UM than OM (because it is not required to inhibit the previous S-R mapping in UM), loss of neural efficiency in implementing cognitive control mechanisms results in a larger N2cc amplitude in UM. This interpretation is also consistent with the reduced level of neural activity after pre-activation within a specific area^45^,^46^. It is also consistent with computational assumptions about pre-activated neural populations taking advantage of populations at rest^47^. As N2cc was larger in UM than in OM, it is possible that cognitive control activity was more strongly modulated by previous activation/inactivation of the mechanisms involved than by the degree of interference that had to be managed.

In accordance with the inhibition of return (IOR) phenomenon, we hypothesized slower RT and N2pc latency^33^ in SM and OM (conditions in which attention is reallocated to the same position in the 50% of trials) than in UM (condition in which attention always comes from a different location, i.e. from the centre of the screen). Specific RT analyses showed absence of IOR. In the present study, we used an inter-stimulus interval (ISI) of around 3000 ms, as is frequently used in Simon task paradigms. Even though IOR was observed with an ISI from 300 ms until 3500 ms, it was stronger at ISI between 300 and 1500 ms. However, at ISI longer than 1500 ms, IOR is mediated by a set of variables such as complexity of the display^48^,^49^. Consistent with RT results, N2pc latency was not longer in SM/OM than in UM. By contrast, the results of the present study showed longer N2pc peak latency in the UM than in the SM and OM conditions. This suggests that attending to a lateralized position (in the n-1 trial) facilitates attending to an upcoming lateralized position (in the n trial), which occurred in SM and OM but not in UM. Therefore, these results are consistent with attentional facilitation in the “n trial” when executing similar attentional sets of operations regarding the “n-1” trial (Hypothesis 4) and suggests the importance of designing specific experimental designs to provide further support for this view.

The larger N2cc amplitude in UM may be related to greater allocation of cognitive control mechanisms to compensate for delays in visuospatial attention processes. Although this interpretation cannot be entirely ruled out, it would be inconsistent with dual route models^3^,^5^,^9^ and with findings of studies based on distributional analysis of RTs^50^, which predict that delays in processing the irrelevant dimension (in this case, the target position) lead to decreased interference. However, the dorsolateral prefrontal cortex (DLPFC) probably exerted high-level cognitive control^51^ via DLPFC-dPM connections^52^,^53^. In this context, Stürmer *et al*.^37^ disrupted left DLPFC by delivering repetitive transcranial magnetic stimulation while the participants carried out a Simon task. These authors observed that the Simon effect occurred in c-I sequences (as usually reported) but that a Simon effect of similar size occurred in i-I sequences. Overall, studies of prefrontal-precentral-parietal connectivity may be of interest for investigating the relationships between the neural mechanisms underlying attention, use of strategies and suppression of inappropriate spatial responses.

In summary, in the present study, increased interference from irrelevant spatial information was found to be related to greater cognitive control activity underlying the ancillary monitoring mechanism, which is involved in managing spatial interference in Simon type tasks by preventing erroneous responses based on stimulus position. Moreover, enhanced electrophysiological activity related to the ancillary monitoring mechanism was observed after trials in which the target stimulus was not lateralized, suggesting reduced neural efficiency for managing spatial interference after trials that do not require managing spatial interference. The present findings also suggested attentional facilitation when executing similar sets of attentional shifts in “n” in the “n-1” trial, as it was observed that attending to a lateralized position facilitated attending to another lateralized position and also that attending to a central position facilitated attending to an upcoming central position.

## Additional Information

**How to cite this article:** Cespón, J. *et al*. Cognitive control activity is modulated by the magnitude of interference and pre-activation of monitoring mechanisms. *Sci. Rep.* **6**, 39595; doi: 10.1038/srep39595 (2016).

**Publisher's note:** Springer Nature remains neutral with regard to jurisdictional claims in published maps and institutional affiliations.

## Figures and Tables

**Table 1:** Mean value (and standard error of mean) for each Condition (Same Mappings (SM), Opposite Mappings (OM), and Unrelated Mappings (UM)) for Reaction Time (RT, in milliseconds); Number of Errors (NE); latencies and amplitudes of negativity posterior contralateral -N2pc- (PO7/PO8) and negativity central contralateral -N2cc- (C3/C4), peak onset and peak latency of lateralized readiness potential –LRP- (C3/C4).

	SM	OM	UM
RT	391 (17.5)	406 (19.6)*	395 (17.5)*•
NE	3.9 (1.1)	6.3 (1.3)*	4.5 (1.1)•
N2pc latency	211 (5.72)	216 (4.49)	236 (4.91)*•
N2pc amplitude	−1.40 (0.47)	−1.55 (0.50)	−2.03 (0.40)
N2cc latency	225 (6.56)	239 (8.09)	237 (7.17)
N2cc amplitude	−0.75 (0.19)	−1.19 (0.27)*	−1.69 (0.28)*•
LRPs onset	211 (10.1)	215 (11.1)	226 (9.7)
LRPs peak latency	352 (13.0)	415 (13.1)*	376 (14.7)*•

Asterisks (*) in OM and UM columns indicate differences between OM and SM and differences between UM and SM, respectively. Circles (•) indicate differences between UM and OM.
